# The Association of Periodontitis and Stroke History: Exploring the Role of Hypertension in a Cross‐Sectional NHANES Analysis

**DOI:** 10.1155/ijod/8825352

**Published:** 2026-03-02

**Authors:** Shi-Yin Jin, Qing Liu, Xiao-Qing Cao, Dian-Wei Wu, Yun-Fan Cai, Li-Na Niu, Wen Qin, Tao Ye

**Affiliations:** ^1^ State Key Laboratory of Oral and Maxillofacial Reconstruction and Regeneration, School of Stomatology, The Fourth Military Medical University, Xi’an, 710032, Shaanxi, China, fmmu.edu.cn; ^2^ The Third Affiliated Hospital of Henan Medical University, Xinxiang, 453000, Henan, China; ^3^ Department of neurology, Air Force Hospital of Eastern Theater Command, Nanjing, Jiangsu, China

**Keywords:** cross-sectional analysis, hypertension, inflammation, national health and nutrition examination survey (NHANES), periodontitis, stroke

## Abstract

**Background:**

Periodontitis, a chronic inflammatory disease, and stroke, a leading cause of death and disability, share common risk factors and inflammatory pathways. However, the role of key mediators such as hypertension in this relationship remains to be fully elucidated.

**Methods:**

This cross‐sectional analysis was conducted using data from the NHANES 2009–2014. Participants aged 30 years and older with complete periodontal and stroke data were included (*n* = 2,783). Periodontitis was classified according to standard NHANES protocols. Stroke history was self‐reported. Survey‐weighted multivariable logistic regression was used to assess the periodontitis‐stroke association. Mediation analysis was performed to quantify the indirect association mediated through hypertension.

**Results:**

In unadjusted models, periodontitis was significantly associated with stroke (OR: 5.69; 95% CI: 2.24–20.61). After full adjustment, this association was no longer statistically significant (OR: 1.48; 95% CI: 0.53–5.67). Mediation analysis revealed a significant indirect association of periodontitis on stroke through hypertension (indirect association coefficient = 0.3685, 95% CI: 0.085–0.868), accounting for 31.30% of the total association. The direct association was not significant.

**Conclusions:**

Hypertension was identified as a significant mediator in the relationship between periodontitis and stroke. The impact of periodontitis on stroke may operate primarily through its association with blood pressure, highlighting the importance of integrated management of periodontal and cardiovascular health.

## 1. Introduction

Periodontitis is a chronic inflammatory disease characterized by the progressive destruction of tooth‐supporting structures, including the periodontal ligament and alveolar bone. The condition manifests through gingival inflammation, clinical attachment loss (AL), increased probing depths (PDs), and alveolar bone resorption [1]. As one of the most prevalent chronic diseases worldwide, periodontitis affects ~11.2% of the global population and represents a significant public health burden [2]. The disease’s impact extends beyond oral health, with mounting evidence suggesting associations with various systemic conditions [3].

Stroke, the second‐leading cause of death globally, affects ~795,000 Americans annually with substantial healthcare costs and disability burden [4, 5]. The potential association between periodontitis and stroke has gained scientific interest due to shared biological pathways involving chronic inflammation, bacterial translocation, and common risk factors [6, 7].

Several mechanisms may link periodontitis to stroke. Chronic periodontal inflammation elevates systemic inflammatory markers, contributing to endothelial dysfunction and atherosclerosis [8]. Periodontal pathogens like *P. gingivalis* can enter systemic circulation, potentially triggering vascular inflammation and plaque instability [9]. Additionally, both conditions share multiple risk factors, including age, smoking, diabetes, socioeconomic status, and notably, hypertension [7, 10]. Among these shared risk factors, the relationship with hypertension deserves particular attention due to its dual role as both a consequence of periodontal inflammation and the most potent modifiable risk factor for stroke.

Hypertension represents a critical mediator in the periodontitis‐stroke pathway. Beyond being a shared risk factor, chronic periodontal inflammation may actively contribute to blood pressure elevation through endothelial dysfunction, increased arterial stiffness, and activation of the renin–angiotensin system [11]. Meta‐analytic evidence demonstrates that individuals with periodontitis have significantly higher blood pressure and increased hypertension prevalence compared to periodontally healthy individuals [12]. Given that hypertension accounts for ~50% of stroke risk, understanding how periodontitis may both share and potentially exacerbate this crucial risk factor has profound implications for stroke prevention strategies [13].

Previous studies have been limited by inadequate confounder control and lack of standardized periodontal assessments. The National Health and Nutrition Examination Survey (NHANES) addresses these limitations through comprehensive periodontal examinations, detailed health assessments, and a nationally representative sampling design.

Therefore, this study investigates the association between periodontitis and stroke using NHANES 2009–2014 data. We aimed to: (1) examine the relationship between periodontitis and stroke in US adults after adjusting for potential confounding factors and (2) investigate which factors mediate the periodontitis‐stroke association, with particular focus on hypertension.

## 2. Materials and Methods

### 2.1. Study Population

We conducted a cross‐sectional analysis using data from the NHANES 2009–2014 cycles. All data and materials are publicly available on the National Center for Health Statistics website (https://www.cdc.gov/nchs/nhanes/index.htm). For this analysis, we included participants aged 30 years and older with complete periodontal examinations and at least one remaining tooth. Participants with missing data on key variables (periodontal status, stroke history, and essential covariates) were excluded from the analysis.

### 2.2. Assessment of Periodontal Disease

Periodontal examinations were conducted by trained dental examiners following standardized NHANES protocols [14]. The classification of periodontal disease is divided into two categories: presence of periodontitis, which includes participants who meet the criteria for any degree of periodontitis; and no periodontitis, which refers to participants not meeting the criteria for any degree [15]. For those classified as having periodontitis, the severity is further graded as follows: mild periodontitis is defined as having ≥2 teeth with clinical AL ≥3 mm and ≥2 teeth with PD ≥4 mm, or any site with PD ≥5 mm; moderate periodontitis is indicated by ≥2 teeth with AL ≥4 mm or ≥2 teeth with PD ≥ 5mm; and severe periodontitis is characterized by ≥2 teeth with AL ≥6 mm and ≥1 tooth with PD ≥5 mm [16].

### 2.3. Assessment of Stroke

Stroke history was ascertained through self‐reported physician diagnosis during the NHANES interview, a method that has been validated in previous epidemiological studies [17]. Participants were classified as having a stroke history if they answered “yes” to the question about whether a doctor had ever told them they had a stroke.

### 2.4. Covariates and Potential Mediators

Demographic variables included age, sex, race/ethnicity, education level, and family income‐to‐poverty ratio. Lifestyle factors included smoking status (never, former, and current) and body mass index (BMI). Clinical covariates included diabetes status (based on self‐report, medication use, or laboratory values), estimated glomerular filtration rate (eGFR, calculated using the 2021 CKD‐EPI equation without race coefficient), fasting glucose, and triglycerides [18]. Hypertension was defined according to the 2017 American Heart Association/American College of Cardiology guidelines as having systolic blood pressure ≥130 mmHg or diastolic blood pressure ≥80 mmHg (based on the average of available measurements), or currently taking antihypertensive medication, or self‐reported physician diagnosis of hypertension [19]. Blood pressure measurements were obtained following standardized NHANES protocols during the examination. White blood cell count (WBC) was considered as a potential inflammatory mediator in the periodontitis‐stroke pathway, as systemic inflammation represents a key mechanistic link between periodontal infection and cardiovascular outcomes [7]. WBC values were standardized (*z*‐scores) for analysis. Other potential mediators examined included hypertension, diabetes, and metabolic factors that may lie on the causal pathway from periodontitis to stroke.

### 2.5. Statistical Analysis

**Figure 1:**
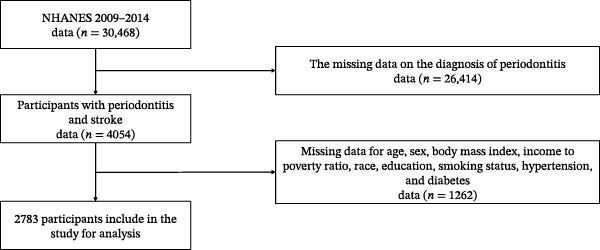
The flowchart of this study.

All analyses accounted for the complex NHANES sampling design using appropriate survey weights (WTMEC2YR), stratification variables (SDMVSTRA), and primary sampling units (SDMVPSU) [20]. For pooled cycles, weights were divided by the number of cycles included to obtain proper population estimates. Population characteristics were presented as means with standard errors for continuous variables and as percentages with standard errors for categorical variables. Group differences were evaluated using the Wilcoxon rank‐sum test for continuous data and the Rao–Scott chi‐squared test tailored for complex survey samples when examining categorical data. The relationship between periodontal disease and stroke was examined using weighted multivariable logistic regression to calculate odds ratios (OR), adjusting for potential confounding factors [21]. Additionally, we used Firth regression to address potential issues with sparse data and better estimate the relationships involving low‐frequency outcomes. The NHANES protocol was approved by the Institutional Review Board of the National Center for Health Statistics, Centers for Disease Control and Prevention, and all participants provided informed consent. A total of 30,468 participants aged 30 years or older were included in this study, 26,414 participants were excluded from the study due to incomplete data on periodontitis and stroke, and an additional 1262 participants were excluded due to missing data on other covariates. Ultimately, 2783 participants were included in the study (Figure 1).

For mediation analysis, we used regression‐based mediation analysis methods implemented in the CMAverse R package to decompose the total association of periodontal disease on stroke into direct and indirect association through potential mediators [22]. The analysis employed loglinear models for the binary outcome and linear models for continuous mediators, with bootstrap confidence intervals (1000 iterations) for statistical inference. We examined multiple potential mediators, including hypertension, diabetes, inflammatory markers, and other risk factors, to identify the key pathways linking periodontal disease to stroke. All statistical analyses were performed in R (version 4.5.1), and statistical significance was defined as *p* < 0.05.

## 3. Results

### 3.1. Demographic, Clinical Characteristics of the Study Population

From NHANES 2009–2014, a total of 2783 participants were included in the analysis, all of whom completed periodontal assessments and relevant health questionnaires. Table 1 summarizes baseline demographic and health‐related characteristics, including the distribution of periodontitis within the study population. The overall age was 50 years (IQR 40–60). The median Family Poverty Income Ratio was 3.10 (IQR 1.47–5.00). Males accounted for 44.66% of the cohort. Overall, 26.63% were ever smokers, and 67.27% were current alcohol consumers. The prevalence of hypertension, diabetes mellitus, and stroke was 57.78%, 12.90%, and 2.73%, respectively. Periodontitis was present in 2203 participants (79.2%), while 580 (20.8%) had no periodontitis.

**Table 1:** Baseline demographic, health‐related characteristics, and periodontitis status of the study population.

Characteristic	Total (*n* = 2,783)^1^	No Periodontitis (*n* = 580)^1^	Periodontitis (*n* = 2,203)^1^	*p*‐Value^2^
Stroke				<0.001
No	2707 (97.27%)	577 (99.48%)	2130 (96.69%)	
Yes	76 (2.73%)	3 (0.52%)	73 (3.31%)	
Age	50.87 ± 13.60	44.93 ± 11.14	53.06 ± 13.77	<0.001
Gender				0.6
Male	1243 (44.66%)	256 (44.14%)	987 (44.80%)	
Female	1540 (55.34%)	324 (55.86%)	1216 (55.20%)	
Race				<0.001
Non‐Hispanic White	592 (21.27%)	92 (15.86%)	500 (22.70%)	
Non‐Hispanic Black	1179 (42.36%)	294 (50.69%)	885 (40.17%)	
Hispanic	609 (21.88%)	93 (16.03%)	516 (23.42%)	
Other/Multirace	403 (14.48%)	101 (17.41%)	302 (13.71%)	
Hypertension	1608 (57.78%)	242 (41.72%)	1366 (62.01%)	<0.001
Diabetes status	359 (12.90%)	35 (6.03%)	324 (14.71%)	0.001
Smoking status				<0.001
Never	2042 (73.37%)	507 (87.41%)	1535 (69.68%)	
Ever	741 (26.63%)	73 (12.59%)	668 (30.32%)	
Education				<0.001
High school or less	589 (21.16%)	39 (6.72%)	550 (24.97%)	
High school graduate	594 (21.34%)	74 (12.76%)	520 (23.60%)	

Some college or more	1600 (57.49%)	467 (80.52%)	1133 (51.43%)	
Alcohol	1872 (67.27%)	417 (71.90%)	1455 (66.05%)	0.4
BMI	29.32 ± 6.92	28.80 ± 6.66	29.51 ± 7.01	0.046
Family poverty income ratio	3.10 (1.47, 5.00)	3.83 ± 1.44	2.73 ± 1.64	<0.001
eGFR	92.09 ± 19.04	95.48 ± 17.00	90.82 ± 19.61	<0.001
WBC	7.32 ± 2.28	7.05 ± 2.13	7.42 ± 2.33	0.042
TG	1.79 ± 1.76	1.67 ± 1.39	1.84 ± 1.88	<0.001
Glu	103.33 ± 37.24	98.08 ± 31.46	105.29 ± 39.01	<0.001

Abbreviations: BMI, Body Mass Index; eGFR, estimated Glomerular Filtration Rate; Glu, Glucose; INDFMPIR, Family Poverty Income Ratio;TG, Triglycerides; WBC, White Blood Cell Count.

^1^Data are *n* (unweighted sample %), Mean ± SD, or Median (IQR) for non‐normally distributed variables.

^2^ *p*‐Values from design‐based tests: Pearson’s X^2^: Rao and Scott adjustment; Design‐based Kruskal–Wallis test.

Hypertension and stroke. Hypertension was more frequent in participants with periodontitis than in those without (62.01% vs. 41.72%; *p* < 0.001; Rao–Scott *χ* ^2^). Stroke was also more common in the periodontitis group (3.31% vs. 0.52%; *p* < 0.001; Rao–Scott *χ* ^2^).

Significant between‐group differences (*p* < 0.05; Rao–Scott *χ* ^2^ for categorical variables; survey‐weighted Wilcoxon rank‐sum test for continuous variables) were observed for age, family Poverty Income Ratio, race/ethnicity, education, BMI, smoking status, hypertension, diabetes mellitus, stroke, eGFR, WBC, triglycerides, and glucose.

### 3.2. Association Between Periodontitis and Stroke

In survey‐weighted logistic regression, stroke events occurred in 73 of 2203 participants with periodontitis and in 3 of 580 without periodontitis (Table 2). Compared with no periodontitis, periodontitis was associated with higher odds of stroke in the unadjusted model (OR: 5.69; 95% CI: 2.24–20.61; *p* < 0.001; survey‐weighted logistic regression), which attenuated after adjusting for age and sex (OR: 2.81; 95% CI: 1.07–10.35; *p* = 0.034; survey‐weighted logistic regression). After further adjustment for race, education, and income (Model 1), the association was no longer statistically significant (OR: 2.06; 95% CI: 0.75–7.79; *p* = 0.175; survey‐weighted logistic regression). In the fully adjusted model additionally controlling for smoking status, alcohol use, hypertension, and diabetes (Model 2), the association remained nonsignificant (OR: 1.48; 95% CI: 0.53–5.67; *p* = 0.486; survey‐weighted logistic regression), indicating no independent association between periodontitis and stroke after adjusting for age, sex, race, education, income, smoking status, alcohol use, hypertension, and diabetes.

**Table 2:** Association between periodontitis and stroke events in survey‐weighted logistic regression models.

Variable	Number of subjects	Number of stroke	Unadjusted model		Age, sex‐adjusted model		Model 1		Model 2	
			OR (95% CI)	*p*‐Value	OR (95% CI)	*p*‐value	OR (95% CI)	*p*‐Value	OR (95% CI)	*p*‐Value
No periodontitis	580	3	1.00 (ref)	<0.001	1.00 (ref)	0.034	1.00 (ref)	0.175	1.00 (ref)	0.486
Periodontitis	2203	73	5.69 (2.24, 20.61)		2.81 (1.07, 10.35)		2.06 (0.75, 7.79)		1.48 (0.53, 5.67)	

*Note:* Model 1: Adjusted for age, sex, race, education, and income. Model 2: Adjusted for Model 1 variables plus smoking status, alcohol use, hypertension, and diabetes.

Abbreviations: CI, confidence interval; OR, odds ratio.

### 3.3. Subgroup Analyses of the Association Between Periodontitis and Stroke

In survey‐weighted subgroup analyses (Table 3), findings were broadly consistent with the main results. In the fully adjusted model (Model 2, controlling for age, sex, race/ethnicity, education, income, smoking, alcohol use, hypertension, and diabetes), no age stratum showed a statistically significant association between periodontitis and stroke, and no clear age gradient was evident. By sex, the association appeared stronger among females, whereas estimates for males were more modest and imprecise; however, wide confidence intervals across both strata limit definitive inference. Stratification by hypertension suggested a higher odds of stroke among participants with hypertension, but neither the hypertensive nor the nonhypertensive groups reached statistical significance. Similarly, the association was detectable among participants without diabetes, while estimates among those with diabetes were highly imprecise; neither subgroup met conventional significance thresholds. It is worth noting that certain subgroups, particularly those aged <45 years, exhibited wide confidence intervals due to limited sample size, warranting cautious interpretation of these estimates.

**Table 3:** Survey‐weighted subgroup analyses of periodontitis and stroke by age, sex, hypertension, and diabetes status.

Variable	Subgroup	Number of stroke /total	Unadjusted		Age, sex‐adjusted		Model 1		Model 2	
			OR (95% CI)	*p*‐Value	OR (95% CI)	*p*‐Value	OR (95% CI)	*p*‐Value	OR (95% CI)	*p*‐Value
Age	<45 years	4/1024	4.52 (0.48–600.04)^a^	0.220	4.40 (0.46–588.00)^a^	0.235	5.57 (0.49–776.38)^a^	0.190	3.45 (0.32–468.81)^a^	0.351
	45–64 years	29/1165	2.85 (0.85–17.76)	0.155	2.50 (0.73–15.73)	0.217	1.94 (0.53–12.55)	0.390	1.54 (0.40–10.12)	0.581
	≥65 years	43/594	3.11 (0.65–55.90)	0.268	2.81 (0.57–50.69)	0.317	2.66 (0.51–48.90)	0.351	2.54 (0.49–46.73)	0.373
Gender	Male	30/1243	3.71 (1.11–23.06)	0.075	1.65 (0.45–10.61)	0.513	1.06 (0.26–7.22)	0.942	0.69 (0.15–4.86)	0.657
	Female	46/1540	12.41 (2.70–220.35)	0.013	6.44 (1.36–115.39)	0.068	6.48 (1.33–117.18)	0.070	4.75 (0.95–86.33)	0.133
Hypertension	No	8/1175	6.94 (0.86–897.91)^a^	0.075	4.47 (0.51–587.44)^a^	0.212	5.64 (0.60–754.20)^a^	0.152	3.69 (0.37–496.65)^a^	0.312
	Yes	68/1608	3.98 (1.46–16.37)	0.020	2.34 (0.83–9.76)	0.161	1.99 (0.68–8.49)	0.268	1.56 (0.52–6.71)	0.482
Diabetes	No	53/2424	4.94 (1.81–20.36)	0.007	2.41 (0.85–10.15)	0.149	2.15 (0.72–9.26)	0.225	3.44 (1.21–14.51)	0.044
	Yes	23/359	5.53 (0.74–708.16)^a^	0.114	4.02 (0.51–518.54)^a^	0.235	3.89 (0.48–504.62)^a^	0.253	4.35 (0.54–563.49)^a^	0.206
BMI	Underweight (<18.5)	1/38	—	—	—	0.619	—	—	—	—
	Normal (18.5–24.9)	22/741	6.45 (1.34–116.10)	0.069	2.95 (0.57–54.20)	0.304	2.15 (0.36–41.52)	0.485	1.57 (0.24–30.99)	0.685
	Overweight (25.0–29.9)	30/922	7.74 (1.64–138.44)	0.045	3.75 (0.74–68.44)	0.204	2.90 (0.55–53.67)	0.313	2.18 (0.40–40.83)	0.465
	Obese (≥30.0)	23/1082	5.67 (1.18–101.82)	0.091	2.93 (0.58–53.53)	0.303	3.42 (0.64–63.42)	0.245	2.30 (0.41–43.26)	0.437
Smoking	Never	45/2042	4.73 (1.71–19.57)	0.010	1.66 (0.56–7.12)	0.417	1.37 (0.44–6.01)	0.624	3.01 (1.05–12.74)	0.073
	Ever/former	31/741	7.26 (1.01–923.30)^a^	0.049	3.53 (0.46–455.09)^a^	0.287	4.50 (0.55–584.91)^a^	0.198	6.33 (0.82–814.76)^a^	0.086
Alcohol	Nondrinker	32/911	14.83 (2.08–1881.41)^a^	0.002	5.63 (0.74–722.90)^a^	0.113	5.63 (0.72–726.57)^a^	0.119	3.92 (0.49–508.84)^a^	0.249
	Drinker	44/1872	4.00 (1.45–16.59)	0.021	2.30 (0.80–9.70)	0.176	1.79 (0.58–7.80)	0.365	1.34 (0.42–5.93)	0.655

*Note*: Estimates for subgroups with limited sample sizes (e.g., Age <45 years) exhibit wide confidence intervals and should be interpreted with caution due to lower precision. Model 1: Adjusted for age, sex, race, education, and income. Model 2: Adjusted for Model 1 variables plus smoking status, alcohol use, hypertension, and diabetes.

Abbreviations: CI, confidence interval; OR, odds ratio.

^a^Analysis conducted using Firth logistic regression with bias correction for rare events.

### 3.4. Mediation Analysis of Hypertension

**Figure 2:**
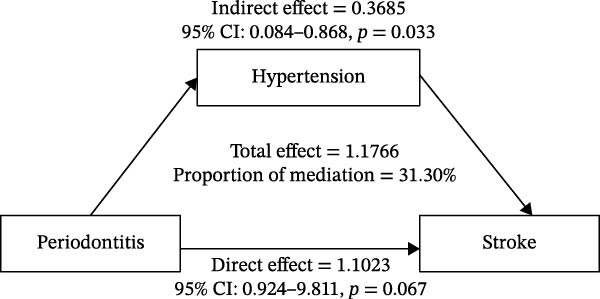
A proposed associative pathway model among periodontitis, hypertension, and stroke history, based on cross‐sectional data.

To explore the mechanism connecting periodontitis to stroke, a mediation analysis was performed with hypertension as the proposed mediator (Figure 2). Notably, no mediating association was identified for the other variables. The analysis revealed a significant pathway from periodontitis to hypertension and from hypertension to stroke. The results demonstrated a significant indirect association of periodontitis on stroke through hypertension (indirect association coefficient = 0.3685, 95% CI: 0.085–0.868, *p* = 0.033). This indirect pathway accounted for 31.30% of the total association. In contrast, the direct association of periodontitis on stroke, after controlling for hypertension (path c’), was not statistically significant (direct association coefficient = 1.1023, 95% CI: 0.924–9.811, *p* = 0.067). Given that the direct association was nonsignificant while the indirect association was significant, these findings are consistent with complete mediation.

## 4. Discussion

In this study, we explored the relationship between periodontitis and the risk of stroke using data from NHANES 2009–2014 comprising 2783 adults. Our discussion focuses on three core findings. First, our analysis indicated a significant association between periodontitis and stroke in unadjusted models, which exhibited an OR of 5.69 (95% CI: 2.24–20.61). However, after adjusting for potential confounders, including sociodemographic variables, lifestyle factors, and comorbidities, this association became nonsignificant. The fully adjusted model resulted in a nonsignificant OR of 1.48 (95% CI: 0.53–5.67), indicating that periodontitis may not be independently associated with stroke history in this population when accounting for hypertension. Second, hypertension emerged as a key variable strongly linked to both conditions. It absorbed a significant portion of the variance, suggesting it plays a central role in the comorbidity profile of these patients. Third, our mediation analysis revealed that hypertension serves as a significant mediator in the periodontitis‐stroke relationship, accounting for ~31.30% of the total association. This identifies hypertension as an important node in the associative network, but longitudinal studies are required for verification. This finding suggests that the impact of periodontal health on cerebrovascular events may operate primarily through its influence on blood pressure regulation rather than through direct mechanisms.

Our results align with several large epidemiological studies that have reported a diminished association between periodontitis and stroke after adjusting for cardiovascular risk factors. For instance, a meta‐analysis conducted by Leira et al. [23] found that while crude estimates indicated a strong relationship, the associations weakened significantly when controlling for potential confounders. Similarly, Lafon et al. [24], in a prospective cohort study, revealed that the association was no longer significant after adjustment for traditional risk factors. Conversely, some studies have shown conflicting conclusions, such as those by Grau et al. [25] and Chen et al. [26], which demonstrated significant associations after adjusting for certain covariates. These differences may stem from variations in study design, definitions of periodontal disease, and the extent of confounding factors accounted for. Our comprehensive adjustment strategy, particularly the inclusion of hypertension as a potential mediator, allows us to more accurately assess the independent association of periodontitis with stroke. This finding provides a clearer perspective on the relationship between periodontal health and cardiovascular health.

The role of hypertension as a mediator between periodontitis and stroke highlights the importance of addressing blood pressure when managing periodontal disease. Chronic periodontal inflammation can initiate systemic inflammatory responses, resulting in increased levels of pro‐inflammatory markers such as C‐reactive protein and interleukin‐6, which are known to contribute to hypertension [27]. Furthermore, the direct invasion of periodontal pathogens into the vascular system can exacerbate hypertension and increase stroke vulnerability [28]. This mechanistic insight underscores the potential benefits of periodontal interventions that may indirectly lower stroke risk by improving hypertension outcomes. Studies have indicated that periodontal therapy can lead to significant reductions in blood pressure, making it a vital consideration in managing patients with both periodontal disease and hypertension [29]. This bi‐directional relationship suggests that healthcare providers should integrate periodontal assessments and treatment into cardiovascular risk management protocols to mitigate both hypertension and stroke risk.

Our findings underscore the need for an interdisciplinary approach to cardiovascular health that includes dental and periodontal care. These results indicate that while periodontal disease may not independently be associated with stroke, the significant mediation through hypertension suggests that enhancing periodontal health could have important repercussions for managing hypertension and preventing cerebrovascular events [30]. Public health initiatives could focus on raising awareness of the links between periodontal health and hypertension, encouraging individuals to seek preventive dental care as part of comprehensive cardiovascular health strategies [31]. Targeting both conditions simultaneously may prove beneficial and warrants further research into integrative health models.

This study is not without limitations. The cross‐sectional design precludes any inference of causality, and the temporal sequences among periodontitis, hypertension, and stroke cannot be established. Therefore, we cannot rule out reverse causality; for instance, post‐stroke motor impairment or cognitive decline might lead to compromised oral hygiene and subsequent periodontitis. Crucially, while the mediation analysis statistically identified an indirect associative pathway through hypertension, the implied causal direction (periodontitis → hypertension → stroke) cannot be confirmed by the concurrent measurement of all variables. Therefore, the reported proportion mediated should be interpreted as a descriptive estimate of the associative structure under a specific statistical model, not as a measure of causal effect. Furthermore, the reliance on self‐reported stroke diagnoses may introduce misclassification bias; however, previous validations have demonstrated acceptable accuracy for such self‐reports. Additionally, despite thorough adjustment for potential confounding variables, residual confounding from unmeasured factors remains a possibility. The selection criteria, requiring at least one remaining tooth, may have resulted in the exclusion of individuals with more severe periodontal disease, affecting the generalizability of our findings.

## 5. Conclusion

In conclusion, our analysis of NHANES 2009–2014 data revealed no independent association between periodontitis and stroke after comprehensive adjustment for confounding factors. However, mediation analysis suggested a statistical pattern where hypertension accounted for a significant portion of the association between periodontitis and stroke history. While these findings suggest that periodontal health may interact with cardiovascular risk primarily through blood pressure regulation, the cross‐sectional design limits causal inference. Therefore, we strongly advocate for future prospective cohort studies to rigorously investigate their potential temporal sequences and causal relationships. Clinically, these results support an integrated approach where managing periodontal health is considered part of the broader strategy for hypertension control and stroke prevention.

## Author Contributions

**Shi-Yin Jin**: conceptualization, methodology, investigation, formal analysis, writing. **Qing Liu**: investigation, data curation, resources, validation. **Xiao-Qing Cao**: methodology, investigation. **Dian-Wei Wu**: conceptualization, writing. **Yun-Fan Cai**: investigation, writing. **Li-Na Niu**: conceptualization, supervision. **Wen Qin**: conceptualization, methodology, formal analysis, supervision, project administration, funding acquisition, writing. **Tao Ye**: conceptualization, methodology, formal analysis, supervision, project administration, funding acquisition, writing. All authors contributed to the study conception and design.

## Funding

This work was supported by the National Natural Science Foundation of China (Grants 82501152 and 82571121) and the Project of State Key Laboratory of Oral & Maxillofacial Reconstruction and Regeneration (Grant 2024QN02).

## Disclosure

All authors read and approved the final manuscript.

## Ethics Statement

The authors have nothing to report.

## Consent

The authors have nothing to report.

## Conflicts of Interest

The authors declare no conflicts of interest.

## Data Availability

The NHANES data is publicly available de‐identified data from the National Center for Health Statistics, and thus, the work was deemed to be exempt by the Institutional Review Board of the University of Massachusetts Boston.
